# The Antioxidant Activity of Atomized Extracts of the Leaves and Stems of *Cnidoscolus diacanthus* (Pax & K. Hoffm.) J.F. Macbr. from Peru and Their Effect on Sex Hormone Levels in Rats

**DOI:** 10.3390/molecules29194554

**Published:** 2024-09-25

**Authors:** Johnny Aldo Tinco-Jayo, León Fernando Pérez-Chauca, Nancy Victoria Castilla-Torres, Edwin Carlos Enciso-Roca, Diana Taboada-Huaman, Litman Nuñez-Soto, Luis Uriel Moscoso-García, Jorge Luis Arroyo-Acevedo, Enrique Javier Aguilar-Felices, Oscar Herrera-Calderon

**Affiliations:** 1 Department of Human Medicine, Faculty of Health Sciences, Universidad Nacional de San Cristobal de Huamanga, Portal Independencia 57, Ayacucho 05003, Peru; johnny.tinco@unsch.edu.pe (J.A.T.-J.); nancy.castilla@unsch.edu.pe (N.V.C.-T.); edwin.enciso@unsch.edu.pe (E.C.E.-R.); enrique.aguilar@unsch.edu.pe (E.J.A.-F.); 2 Academic Department of Chemical Engineering, Universidad Nacional de San Cristóbal de Huamanga, Portal Independencia 57, Ayacucho 05003, Peru; leon.perez@unsch.edu.pe; 3 Professional School of Pharmacy and Biochemistry, Faculty of Health Sciences, Universidad Nacional de San Cristobal de Huamanga, Portal Independencia 57, Ayacucho 05003, Peru; diana.taboada.20@unsch.edu.pe (D.T.-H.); litman.nunez.20@unsch.edu.pe (L.N.-S.); 4 Academic Department of Biological Sciences, Faculty of Biological Sciences, Universidad Nacional de San Cristobal de Huamanga, Portal Independencia 57, Ayacucho 05003, Peru; luis.moscoso@unsch.edu.pe; 5 Department of Dynamic Sciences, Faculty of Medicine, Universidad Nacional Mayor de San Marcos, Av. Miguel Grau 755, Lima 15001, Peru; jarroyoa@unmsm.edu.pe; 6 Department of Pharmacology, Bromatology and Toxicology, Faculty of Pharmacy and Biochemistry, Universidad Nacional Mayor de San Marcos, Jr. Puno 1002, Lima 15001, Peru

**Keywords:** huanarpo hembra, sexual hormones, fertility plants, Peruvian plants, ethnopharmacology, antioxidant agents, fecundity

## Abstract

In this research, we aimed to determine the antioxidant activity of an atomized extract of *Cnidoscolus diacanthus* (Pax & K. Hoffm.) J.F. Macbr., known in Peru as “huanarpo hembra”, and its effect on sex hormone levels. Its phytochemical profile was determined using liquid chromatography–mass spectrometry (LC–MS), while its total phenol content (TPC) and total flavonoids (TFs) were determined using the Folin–Ciocalteu method and the aluminum chloride method. Its antioxidant activity was determined using 2,2-diphenyl-1-picrylhydrazyl (DPPH), the radical 2,2-azino-bis-3-ethylbenzthiazolin-6 sulfonic acid (ABTS), and ferric-reducing antioxidant power (FRAP). The biological activity of *C. diacanthus* and its effect on sexual hormones were determined in Holtzman rats of both sexes. Phytochemical analysis revealed the presence of flavonoids and phenolic compounds in its leaves and stems, mainly rutin, quercetin, chlorogenic acid, and genistein. However, the stem extract contained higher total phenol (464.38 ± 4.40 GAE/g) and flavonoid (369.17 ± 3.16 mg QE/g of extract) contents than the leaf extract (212.38 ± 3.19 mg GAE/g and 121.49 ± 2.69 mg QE/g). For DPPH, ABTS, and FRAP, the Trolox-equivalent antioxidant capacity (TEAC) was 597.20 ± 5.40 µmol/g, 452.67 ± 5.76 µmol/g, and 535.91 ± 1.56 µmol/g, respectively, for the stems, while for the leaves, it was 462.39 ± 3.99 µmol/g, 202.32 ± 5.20 µmol/g, and 198.13 ± 1.44 µmol/g, respectively. In terms of the values for hormonal levels, at a dose of 100 mg/kg of the extract, testosterone levels of 1.430 ng/mL (with the leaf extract) and 1.433 ng/mL (with the stem extract), respectively, were found in the male rats. Regarding estradiol levels, in the female rats, these were 10.425 ng/mL (leaf extract) and 8.775 ng/mL (stem extract), while their levels of luteinizing hormone were 0.320 mIU/mL (leaf extract) and 0.273 mIU/mL (stem extract). For the follicle-stimulating hormone, levels of 0.858 mIU/mL (leaf extract) and 0.840 mIU/mL (stem extract) were found in the female rats, and levels of 0.220 mIU/mL (leaf extract) and 0.200 mIU/mL (stem extract) were found in the male rats. It is concluded that the *C. diacanthus* stem extract had a greater antioxidant capacity than the leaf extract, while both extracts had a superior effect on the sex hormone levels in the female rats compared to the male rats.

## 1. Introduction

Infertility, defined as the inability to conceive after one year or more of consistent and unprotected sexual intercourse, is a medical condition that affects both male and female reproductive systems [1]. Indeed, it occurs in approximately 10–15% of people of reproductive age worldwide. However, in certain regions of the developing world, these rates exceed 30% due to the increased prevalence of sexually transmitted infections and diseases, unsafe abortions, and pelvic infections postpartum. In women, infertility can be caused by various conditions, including hyperprolactinemia and ovulation-related issues [2]. Ovulatory dysfunction, which occurs in 25 to 50% of cases, is primarily caused by hormonal issues associated with the dysfunction or malfunction of the hypothalamic–pituitary–ovarian axis [3]. Meanwhile, in men undergoing fertility evaluations, low levels of testosterone or high luteinizing hormone (LH) levels have been detected in about 30% of patients [4]. Estrogen and progesterone are the primary sex hormones responsible for female sexual characteristics, with their production and release stimulated by pituitary hormones, specifically the follicle-stimulating hormone (FSH) and luteinizing hormone (LH). On the other hand, in men, LH is the primary hormone that regulates testosterone production [5], and these sex hormones also play other crucial roles in maintaining overall health and preventing ill health.

Generally, the use of natural supplements in fertility management is associated with their antioxidant activity. Some systematic reviews have referred to well-known plant species like *Zingiber officinale*, *Cinnamomum zeylanicum*, and *Phoenix dactylifera* improving sperm quality and testosterone production in men on this basis [6]. Equally, in women, certain reproductive disorders have been modulated and their hormonal balance maintained using antioxidant compounds from garlic, such as allicin and ajoene [7]. Phenolic compounds are the major class of secondary metabolites that have been studied in antioxidant supplements, some of which are considered phytoestrogens, like isoflavones (genistein, daidzein, and glycitein), which bind to estrogen receptors [8]. However, the effects of flavonoids on sex-steroid-dependent activities remain unknown. Recently, natural products with high free radical scavenging capacities have also received increased attention as potential alternatives for treating oxidative stress and hormonal irregularities given their ability to mimic endogenous estrogen activity, inhibit hormone activity, and modulate the production of hormones, including that of sex hormones [9].

**Figure 1:**
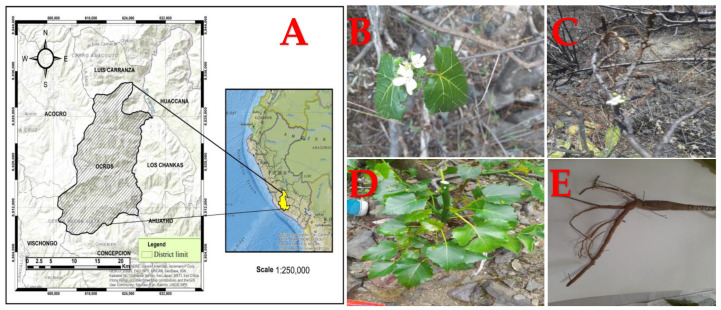
(**A**) Geographical location of the Ocros district. (**B**) Flowers, (**C**) stems, (**D**) leaves, and (**E**) roots from *Cnidoscolus diacanthus* (Pax. & Hoffm.) Macbr.

Known as “huanarpo hembra” in Spanish, *Cnidoscolus diacanthus* (Pax & K. Hoffm.) J.F. Macbr. is a monoecious shrub approximately 1.5 to 2 m high and covered in large stinging hairs (Figure 1). More than 99 species belonging to the *Cnidoscolus* genus are distributed across the Americas, from the United States to Argentina, while its greatest diversity is found in Mexico and Brazil. In Peru, this species grows in Ayacucho at 2800 m altitude and has traditionally been used by infertile women [10]. Although there are currently no reports on its chemical characterization, its traditional uses have pertained to its aphrodisiac properties, especially in the context of female infertility. In another study, other species from the *Cnidoscolus* genus, like *C. aconitifolius*, were reported to act as contraceptives due to the changes they caused in reproductive hormones in female rats and their negative impact on follicle growth and ovulation [11]. Many bioactive compounds of different kinds have been discovered in this genus and linked to anti-inflammatory, hypoglycemic, antimicrobial, hepatoprotective, cardioprotective, and vasoconstrictor effects [12]. Hence, our aim was to evaluate the total phenol and total flavonoid contents in the leaves and stems of this plant, as well as their antioxidant capacity, using antioxidant tests (DPPH, ABTS, and FRAP) and to ultimately determine their effects on sex hormone levels in rodents of both sexes using fluorescence immunoassay techniques.

## 2. Results

### 2.1. LC–MS Analysis of the Atomized Extract

**Figure 2:**
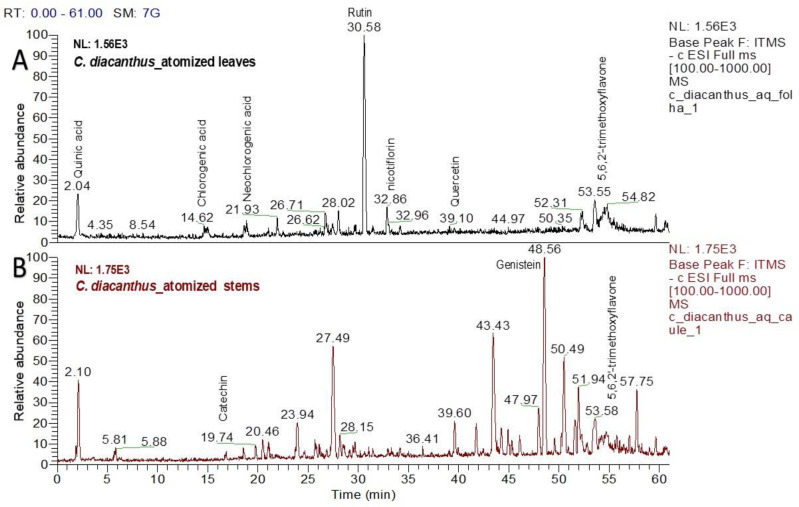
Chromatograms of the atomized extracts of the leaves (**A**) and stems (**B**) of *C. diacanthus*. (Scan rang *m*/*z* 100–1000).

Using LC–MS for the phytochemical analysis, seven compounds were identified in the atomized extract of the leaves, and three compounds were identified in the extract of the stems. Figure 2 shows the chromatograms for each extract, and Table 1 and Table 2 show the names of their chemical structures. In this analysis, flavonoidal structures were the major compounds found and are referenced according to the literature. In detail, the MS^2^ spectra of these putative compounds are shown in the Supplementary Materials (Figures S1–S8).

### 2.2. Determination of Total Phenolic Content and Total Flavonoids

To determine the total phenols and total flavonoids in the atomized *C. diacanthus* leaf and stem extracts, Folin–Ciocalteu and aluminum chloride reagents were used. For quantification of the total phenols, a calibration curve was created using gallic acid as the reference standard, which showed a correlation coefficient of R^2^ = 0.9977 for total phenols and R^2^ = 0.9966 for total flavonoids. In Table 3, we can see that the leaf extract differed significantly (Student’s *t*-test; *p* < 0.0001) from the stem extract in its total phenol and total flavonoid contents.

### 2.3. Determination of the Antioxidant Activity

Table 4 shows the antioxidant activity of the atomized leaf and stem extracts of *C. diacanthus* as assessed using three different assays. For DPPH, the antioxidant activity of the leaf and stem extracts was 67.53% ± 0.25 and 53.20% ± 0.21, respectively. According to the ABTS method, the results were 452.67 ± 5.20 µmol TE/mg for the stems and 202.32 ± 5.76 µmol TE/mg for the leaves; in this case, the increase in the antioxidant capacity was 69.83% ± 0.73 using the stems and 34.63% ± 0.81 using the leaves. Finally, the FRAP was 535.91 ± 1.56 µmol TE/mg for the stems and 198.13 ± 1.44 µmol TE/mg for the leaves. In addition, the stem extract was found to have a greater antioxidant activity using the DPPH, ABTS, and FRAP methods than the leaf extract.

### 2.4. Hormonal Effect of the Atomized Extract of C. diacanthus

#### 2.4.1. Effect on Testosterone

**Figure 3:**
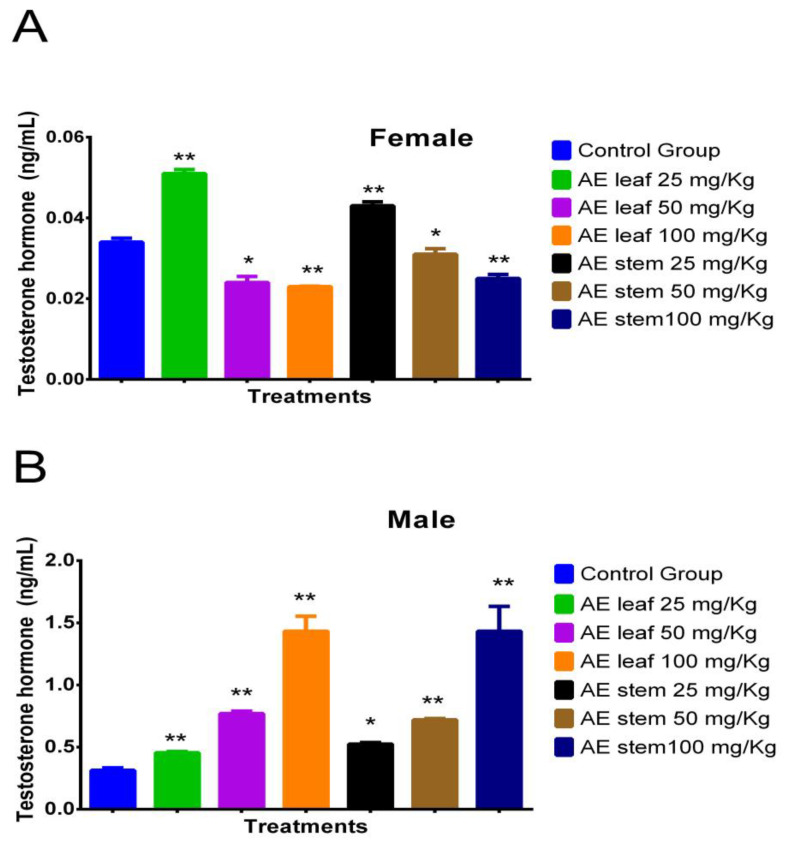
Effect of treatment with atomized extracts of the leaves and stems of *C. diacanthus* on the levels of the hormone testosterone in female (**A**) and male (**B**) Holtzman rats. * *p* < 0.001, ** *p* < 0.0001; Tukey’s test. These results are the average ± SD of three determinations.

Figure 3A shows the effect of atomized extracts of *C. diacanthus*’ leaves and stems on the hormone testosterone in female Holtzman rats. At a dose of 25 mg/kg of the leaf extract, their testosterone levels significantly increased in comparison to those at the other doses tested and those in the control group (*p* < 0.0001). Furthermore, there was no difference between doses of the leaf extracts of 50 mg/kg and 100 mg/kg (*p* = 0.8512) or using the leaf and stem extracts at a dose of 100 mg/kg (*p* = 0.1230). Meanwhile, Figure 3B shows that using 100 mg/kg of the leaf and stem extracts in the male rats caused a significant increase in testosterone, with no significant difference between the effects of the extracts at this dose at a value of 1.433 ± 0.2 ng/mL (*p* > 0.9999). In addition, the results of using these other extracts at different doses significantly differed from those of the control group (distilled water) (0.313 ± 0.01 ng/mL); at a dose of 50 mg/kg, levels of 0.77 ± 0.02 ng/mL and 0.72 ± 0.01 ng/mL were reached using the leaf and stem extracts, respectively, with no significant difference (*p* = 0.9654), while at a dose of 25 mg/kg, levels of 0.455 ± 0.02 ng/mL and 0.525 ± 0.01 ng/mL were reached using the leaves and the stems (*p* = 0.8264).

#### 2.4.2. Effect on Progesterone

**Figure 4:**
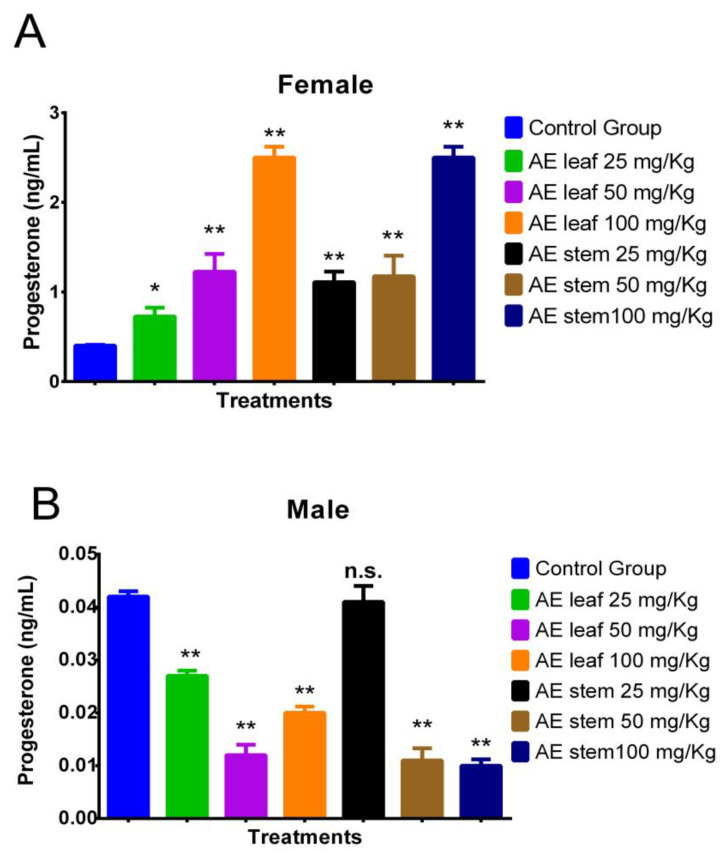
Effect of treatment with atomized extracts of the leaves and stems of *C. diacanthus* on the levels of the hormone progesterone in female (**A**) and male (**B**) Holtzman rats. * *p* < 0.001, ** *p* < 0.0001, n.s. (non-significant “*p*” value); Tukey’s test. The results are the average ± SD of three determinations.

Figure 4A shows how at doses of 100 mg/kg, the atomized extracts of *C. diacanthus*’ leaves and stems increased the levels of the hormone progesterone in female Holtzman rats compared to the levels in the control group (*p* < 0.0001). Furthermore, both extracts caused significant increases, except the leaf extract at a dose of 25 mg/kg. Figure 4B shows the results in the male rats: their progesterone levels decreased under treatment with both extracts, except when using a 25 mg/Kg dose of the stem extract, at which dose the result was not significantly different (*p* = 0.0808) compared to the control group.

#### 2.4.3. Effect on Estradiol (E_2_)

**Figure 5:**
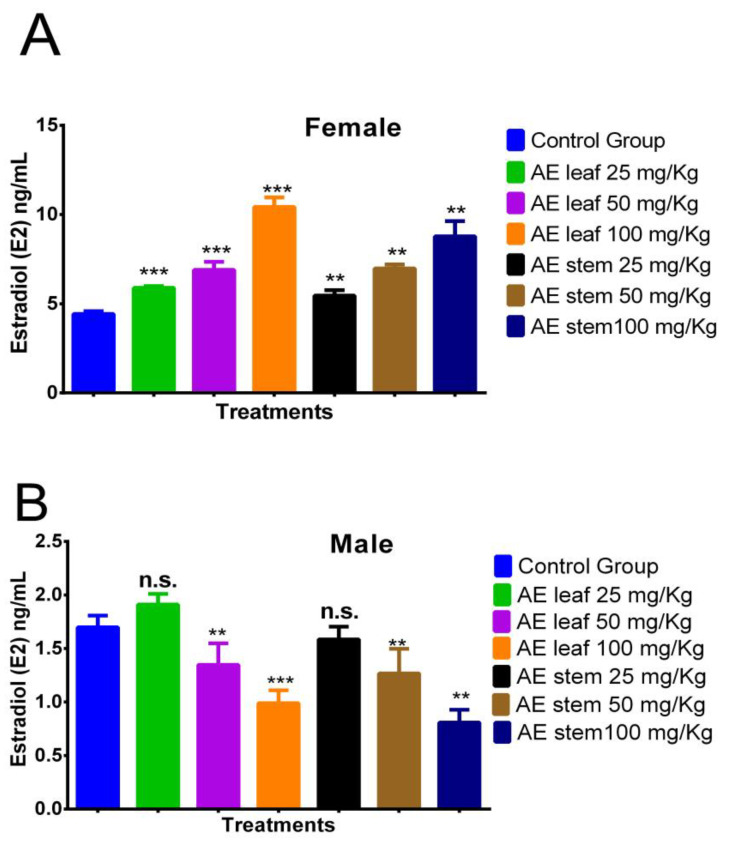
Effect of treatment with atomized extracts of the leaves and stems of *C. diacanthus* on the levels of the hormone estradiol in female (**A**) and male (**B**) Holtzman rats. ** *p* < 0.001, *** *p* < 0.0001, n.s. (non-significant “*p*” value); Tukey’s test. The results are the average ± SD of three determinations.

In Figure 5A, it is evident that the atomized extracts of *C. diacanthus’* leaves and stems increased the estradiol (E_2_) levels in the female rats. The leaf extract induced estradiol levels of 5.893 ± 0.89 ng/mL at a dose of 25 mg/kg, 6.900 ± 0.72 ng/mL at a dose of 50 mg/kg, and 10.425 ± 1.12 ng/mL at a dose of 100 mg/kg, while the same results for the stem extract were 5.450 ± 0.11 ng/mL, 6.975 ± 0.91 ng/mL, and 8.775 ± 0.63 ng/mL, respectively. Furthermore, all the doses tested of the leaf (*p* < 0.0001) and stem extracts (*p* < 0.001) caused higher levels of estrogen than those in the control group, at 4.425 ± 0.52 ng/mL. Meanwhile, in the male rats, Figure 5B reveals the significant decrease found when using 100 mg/Kg of both extracts compared to the levels in the control group (leaves: *p* < 0.0001; stems: *p* < 0.001). In addition, non-significant *p* values were reported for both extracts at doses of 25 mg/Kg.

#### 2.4.4. Effect on Luteinizing Hormone (LH)

**Figure 6:**
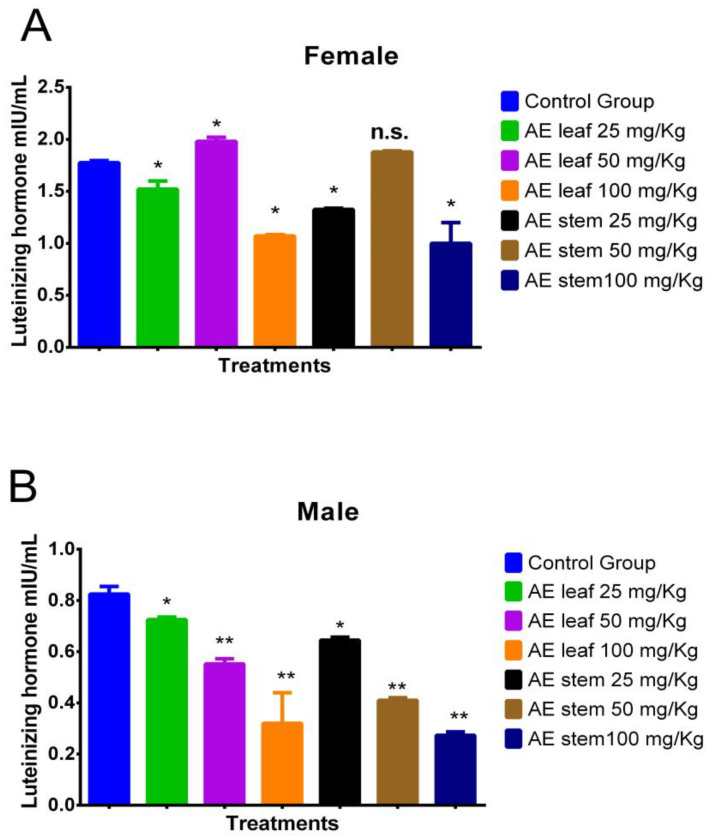
Effect of treatment with atomized extracts of the leaves and stems of *C. diacanthus* on the levels of luteinizing hormone in female (**A**) and male (**B**) Holtzman rats. * *p* < 0.001, ** *p* < 0.0001, n.s. (non-significant “*p*” value); Tukey’s test. The results are the average ± SD of three determinations.

Figure 6A shows the effect of the atomized extracts of *C. diacanthus’* leaves and stems on luteinizing hormone (LH) in female Holtzman rats. As shown in Figure 6A, a significant increase in their LH levels was observed at a dose of 50 mg/kg of the leaf extract compared to the levels in the control group (*p* < 0.001). However, at the other doses, the use of both extracts caused a significant drop in LH levels. Regarding the effect of the atomized extracts on the male rats (Figure 6B), their levels of LH were lowered by both extracts compared to those in the control group, with a dose of 100 mg/kg of both extracts having the best effect in lowering their LH levels (*p* < 0.0001).

#### 2.4.5. Effect on Follicle-Stimulating Hormone (FSH)

**Figure 7:**
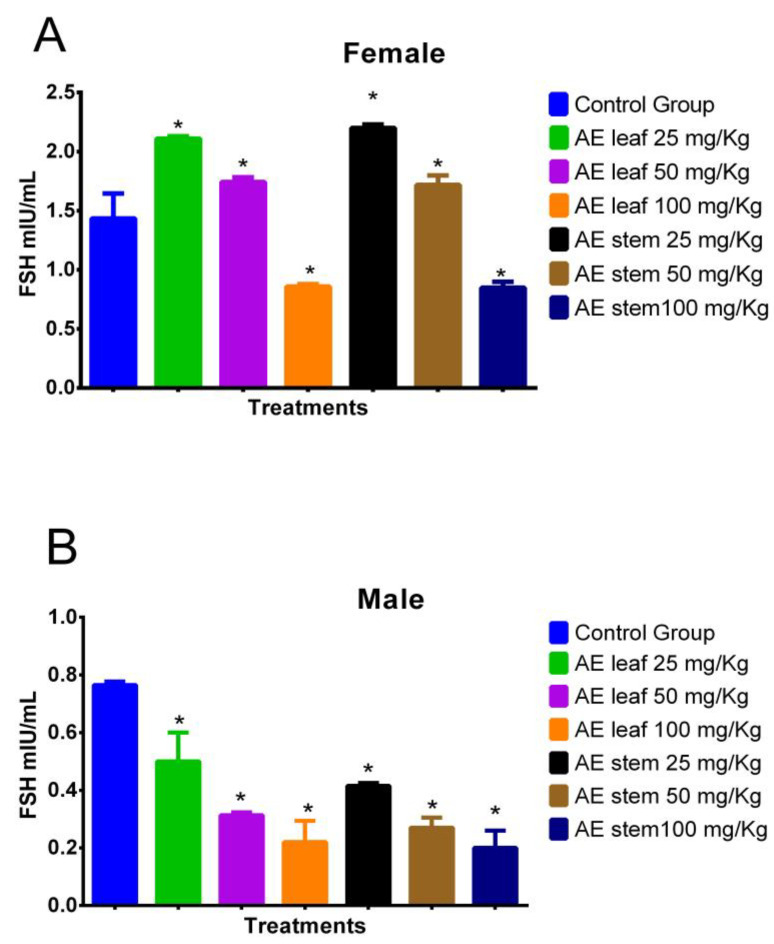
Effect of treatment with the atomized extracts of the leaves and stems of *C. diacanthus* on the levels of follicle-stimulating hormone (FSH) in female (**A**) and male (**B**) Holtzman rats. * *p* < 0.001; Tukey’s test. The results are the average ± SD of three determinations.

Figure 7A shows the effect of treatment with atomized extracts of the leaves and stems of *C. diacanthus* on the levels of follicle-stimulating hormone (FSH) in female Holtzman rats. The results show a significant increase (*p* = <0.0001) at a dose of 100 mg/kg of the leaf and stem extracts in the female rats with respect to the levels in the control group. On the other hand, at a dose of 25 mg/Kg, the levels of this hormone increased to values of 2.110 ± 0.81 mIU/mL using the leaf extract (*p* < 0.0001), and 2.200 ± 0.70 mIU/mL using the stem extract compared to those in the control group (*p* < 0.0001). Meanwhile, in Figure 7B, representing the male rats, it is observed that the leaf and stem extracts caused a significant decrease (*p* < 0.0001) in their FSH levels compared to those in the control group, with the lowest FSH levels in the animals treated with doses of 100 mg/kg (*p* < 0.0001).

## 3. Discussion

Chemical research on various species from the *Cnidoscolus* genus has identified the presence of certain bioactive chemicals in different plant organs such as the leaves, stems, bark, roots, and seeds, with secondary metabolites including flavonoids, triterpenes, tannins, coumarins, and steroids isolated from this genus in phytochemical studies [15]. Regarding the phytochemical determination in this study, the bioactive compounds detected in the atomized extracts of the leaves and stem of *C. diacanthus* have also been reported in other species in the genus, including quercetin, neochlorogenic acid, chlorogenic acid, 5,6,2-trimethoxyflavone, and quercetin 3-rhamnoglucoside in *Cnidoscolus chayamansa* [18]; quinic acid and kaempferol in *Cnidoscolus aconitifolius* [19]; 3-O-glucoside-7-O-rhamnoside (nicotiflorin) in *Cnidoscolus chayamansa* [20]; and also genistein in *Cnidoscolus aconitifolius* [19], in an extract of its stems [21]. In this study, putative identification of these compounds using LC–MS was described for the first time, with commonly encountered phenolic compounds and their derivates found in both extracts.

Regarding quantification of its total phenol and flavonoid contents, our results for *C. diacanthus* differed from those reported by Zambrano et al. [22] for *Cnidoscolus tehuacanensis*, the leaf extract of which had a low content of gallic acid, at 3.023 ± 0.06 mg GAE/g. Medina et al. [23] reported that an aqueous extract of *C. aconitifolius* had a higher content of total phenols (70.61 ± 0.07 mg GAE/100 g of extract), while the ethanolic extract had a higher content of flavonoids (47.76 ± 4.84 g GAE/100 g of extract), flavanones, and dihydroflavonols (70.10 ± 7.29 g/100 g of extract). The difference observed in this research may be attributed to the type of extraction used, which was spray-drying. On the contrary, a dramatic decrease in the phenolic content of some samples has also been reported [24]; this can be directly attributed to the impact of the concentration of maltodextrin used as a stabilizing agent in the process. Additionally, there are indications in the literature that spray-drying might lead to a reduction in the overall phenolic content and antioxidant activity of powdered extracts, suggesting a loss or alteration of phenols during the drying process. Nevertheless, it is crucial to acknowledge that the influence of spray-drying on phenolic activity might differ depending on multiple parameters, including the drying conditions, the phenolic concentration in the raw material, and the specific plant materials used [24].

Loarca-Piña et al. [25] determined the antioxidant activity of *Cnidoscolus chayamansa* leaves using colorimetric DPPH and ABTS assays, observing that methanol extract had a TEAC value of 144.68 ± 13.7 μmol/g extract using DPPH and 214.18 ± 29.4 using the ABTS method. Similar results were obtained for the leaf extract of *C. diacanthus* using the ABTS method. In addition, Zambrano et al. [22] assessed *Cnidoscolus tehuacanensis*’ antioxidant activity using the DPPH assay. Even at the maximum concentration (2 mg/mL), the crude extract showed a poor inhibition percentage of 22.36%± 0.02, while at the minimum concentration (125 µg/mL), the inhibition percentage was 15.40%. Therefore, these reported results suggest that the antioxidant activity of this species is low, although they might have been influenced by the drying method or the type of solvent used in the study. On the other hand, using the DPPH and FRAP methods, Garcia-Rodrigues et al. [26] established the antioxidant activity of *Cnidoscolus chayamansa* leaves in different solvents at a concentration of 33 mg/mL: in the DPPH assay, it was 11.687% ± 1.00 in ethyl acetate, 10.667% ± 0.87 in ethanol, and 10.587% ± 0.36 in n-hexane. Meanwhile, using the FRAP method, 387.167 ± 8.0 (mmol Fe^+2^/L), 254.047 ± 2.63 (mmol Fe^+2^/L), and 239.477 ± 0.73 (mmol Fe^+2^/L) were obtained in the ethyl acetate fraction, the ethanolic extract, and the hexane extract, respectively. On this basis, the solvent used was assumed to have an influence on the antioxidant activity of this species.

Regarding the potentially low antioxidant activity found in atomized extracts, Krishnaiah et al. [27] determined that DPPH radical inhibition and phenolic content significantly decreased when an increased temperature was used during the spray-drying process. Elevated temperatures are generally known to have a negative impact on the composition of phenolic compounds, many of which are thermolabile, resulting in structural damage and the formation of other molecules, ultimately leading to a decrease in antioxidant activity [28]. Unfortunately, in this study, we did not compare how obtaining extracts of this plant using different drying processes affected the antioxidant activity in order to determine whether low antioxidant activity might be linked to the drying process. On the other hand, in the evaluation of its antioxidant profile, the stem extract had a superior effect in the FRAP assay, indicating that its antioxidant mechanism of action involves electron transfer processes [29]. In this case, the antioxidant activity was measured according to its capacity to convert Fe^3+^-tripyridyltriazine into Fe^2+^-tripyridyltriazine.

According to the results on the variation in sex hormones in the rodents, the male rats treated with 100 mg/kg of the stem and leaf extracts had higher testosterone levels. These observed effects may be attributed to the presence of specific flavonoids such as quercetin, which has been found to lead to an increase in testicular and body weight, spermatogenesis, and the production of androgenic hormones (FSH, LH, and testosterone) in prior research [30]. Catechins [31], genistein [32], flavanones, and flavanols like rutin, quercetin [33], and kaempferol [34] increased plasma testosterone in male rodents. In terms of the hormone estradiol (E_2_), both extracts increased the levels of this hormone in the female rats, with the opposite effect observed in the male rats. Their effects might be related to their contents of certain secondary metabolites—for example, flavonoids like isoflavones and flavanones [35] since they have a similar structure to estrogens, with genistein found in the stem extract in this study, and flavones, like 5,6,2′-trimethoxyflavone, which was previously reported in the leaves of *C. nidoscolus*. Slighoua et al. determined an increase in estradiol of between 8.7 and 22.48% in female rats treated with quercetin at a dose of 10 mg/kg [36]. Furthermore, ovariectomized rats treated with rutin showed high estradiol levels [37]. Rodrigues-Landa et al. [38] refer to estradiol, estrone, and estriol as the most abundant circulating estrogens in the human body, with steroidal structures that allow them to interact with estrogen receptors (ERs) and trigger a series of key physiological responses. For instance, increases in 17-β estradiol (E_2_) allow the vagina, uterus, and uterine tubes to reach full development and functionality, as well as stimulating the growth of breast ducts [39]. In addition, plants with estrogenic properties can also influence pituitary action through the peripheral modulation of LH and FSH, decreasing their secretion and disrupting ovulation [40].

Moreover, in this study, we found that the atomized extracts of *C. diacanthus* increased the progesterone, estradiol, and FSH levels at varied doses in the female rats but decreased their luteinizing hormone levels. A similar result was reported by Abedpour et al. [41]. They found that when chlorogenic acid was administrated to female rats, the levels of estradiol, FSH, and progesterone were higher, but the levels of LH were lower than in groups that did not receive chlorogenic acid. Our findings demonstrated that at doses of 25 and 50 mg/kg, the leaf and stem extracts increased the FSH and LH levels in the female rats but decreased the levels of these hormones in the male rats. This observation suggests that the extract altered hormone levels differentially in male and female rats due to their distinct reproductive systems. Some studies have demonstrated that in female rats, plant extracts may stimulate the reproductive system by elevating FSH and LH levels, thereby boosting ovulation and fertility, whereas in males, elevated testosterone levels or hormonal imbalance diminished FSH and LH, thereby would inhibit the sperm production and fertility [42]. In addition, there were also high levels of progesterone and estradiol and high levels of testosterone observed in the female rats at doses of 100 mg/Kg and 25 mg/Kg, respectively, suggesting the latter dose might negatively affect fertility on the basis of high testosterone [43].

Additionally, this species has traditionally been used by women in Ayacucho, Peru, to treat general sexual problems. Some studies have asserted that enhancing hormones like E_2_, LH, and FSH can improve fertility, particularly in women [44]. However, further studies are needed to understand the biological mechanism behind the atomized extracts of *C. diacanthus* causing variations in hormone levels in organisms.

## 4. Materials and Methods

### 4.1. Plant Material

The leaves and stems were collected in Ocros district, Huamanga province, in the department of Ayacucho, Peru (located at a 3125 m altitude), and put into kraft paper bags. They were transferred to the pharmacology laboratory of the Professional School of Pharmacy and Biochemistry at the Universidad Nacional de San Cristobal de Huamanga. Botanical determination of *Cnidoscolus diacanthus* (Pax. & Hoffm.) Macbr., known as “huanarpo hembra”, was carried out at the Museum of Natural History of the Universidad Nacional Mayor de San Marcos (N021-USM-MHN-2022) by Mag. Hamilton Beltrán Santiago.

### 4.2. Sample Preparation

*C. diacanthus* leaves that were in excellent condition were selected and placed onto kraft paper to dry at room temperature in the shade. The stems were also selected, cut crosswise with a knife, and placed onto kraft paper to dry in the same conditions. During the three weeks of drying, the kraft paper was constantly changed, and the samples were turned over to ensure they dried uniformly and did not decompose. Once the samples were completely dry, they were ground mechanically using a manual mill to obtain powder, which was then stored in amber bottles at room temperature.

### 4.3. Obtention of the Atomized Extracts from C. diacanthus

The leaves and stems were processed separately. First, 700 g of the leaves and 800 g of the dried and ground stems were macerated with 1 L of 70% ethanol in separate amber glass containers for 15 days and continuously stirred for 15 min each day. The samples were filtered twice with a vacuum pump. Subsequently, they were concentrated in a rotary evaporator (R300 Buchi, Flawil, Switzerland); then, 1 L of distilled water was added. Finally, the extracts were dried using a spray-dryer (OLT-SD8000B, Ayacucho, Peru) and stored in amber bottles until further use.

### 4.4. Chemical Characterization by LC–MS

#### 4.4.1. Pretreatment

Individually, 10 mg of each sample was transferred into a 2 mL microtube. Subsequently, 1 mL of methanol was introduced into each microtube, followed by sonicating it for 15 min and vertexing it for 30 s. Subsequently, 200 µL of each solution was mixed with 300 µL of methanol and 500 µL of purified water. Then, it was centrifuged at 10,000 RPM for 15 min, and finally, 750 µL of the supernatant was chromatographically analyzed using LC–MS.

#### 4.4.2. Chromatographic Conditions

The chromatographic analysis was conducted using a UHPLC Dionex™ UltiMate™ 3000 system (Waltham, MA, USA) equipped with an ACQUITY UPLC^®^ BEH C18 reversed-phase column (2.1 mm × 150 mm, 1.7 μm, Waters, Milford, MA, USA). The flow rate of the mobile phase was set at 0.2 mL/min. The temperature of the column was maintained at 30 °C, with gradient elution of the mobile phase consisting of a mixture of water containing 0.1% formic acid (A) and methanol (B). The initial conditions were 5% B held for 5 min, ramped to 98% for 55 min, held for 5 min at 95%, and then returned to 5% for 1 min. Diode array detection was performed at wavelengths of 220, 254, 280, and 365 nm.

The MS analysis was conducted using the LCQ Fleet Ion Trap 3D mass spectrometer (ThermoFisher in San Jose, CA, USA), which had an ESI source. The spectra were obtained in negative ion mode using an Ion Trap 3D mass analyzer with a mass resolution of 1000. Extremely pure helium (He) was used as the collision gas, and high-purity nitrogen (N2) was used as the nebulizing gas. The ion spray voltage was set to 5 kV, and the sheath gas (N2) flowed at a rate of 35 arbitrary units. The auxiliary gas (N2) flowed at a rate of 10 arbitrary units. The capillary temperature was set to 400 °C, and the capillary voltage was −33 V. The tube lens voltage was −91.07 V. The data were processed using the Thermo XcaliburTM software version 3.0 (Thermo Fisher Scientific Inc., Waltham, MA, USA) with the Qual Browser, and metabolite annotations were performed with MS-Dial software version 4.70 (Riken, Osaka University, Suita City, Japan) using the MS-Dial metabolomics MPS spectral kit library (available at http://prime.psc.riken.jp/compms/msdial/main.html; last updated on 13 April 2021). In MS scan mode, the data acquisition was conducted using a full scan from *m*/*z* 100 to 1000. The collision energy for the MS^2^ spectra was 35, the width of the precursor ion isolation was 2.0 *m*/*z*, and the activation time was 30 ms.

### 4.5. Determination of the Total Phenolic Content

A volume of 150 µL of the sample (1 mg/mL) was collected and mixed with 2400 µL of distilled water. Subsequently, 150 µL of 0.25 N Folin–Ciocalteu reagent (St. Louis, MO, USA) was added, and the mixture was mixed vigorously for 5 min. The resulting mixture was then left to react for 3 min. Finally, 300 µL of 1 N sodium carbonate (Na_2_CO_3_) solution was added, and the mixture was mixed again for 5 min. This combination was placed in a dark environment at a temperature of 20 °C and left undisturbed for a duration of 2 h. The absorbance measurement was taken using a GENESYS 10 UV-VIS spectrophotometer (Thermo Scientific, Waltham, MA, USA) at a wavelength of 725 nm. The calibration curve was constructed using gallic acid at concentrations of 0.0, 5.0, 10.0, 15.0, 20.0, 25.0, and 30.0 μg/mL. The experiments were conducted three times, and the results are presented as milligrams of the gallic acid equivalents per grams of extract (mg GAE/g of extract) [45].

### 4.6. Determination of the Flavonoid Content

In brief, 500 µL of distilled water and 150 µL of 5% sodium nitrite (NaNO_2_) were added to 500 µL of the sample (1 mg/mL); the subsequent mixture was vortexed for 5 min and then allowed to react for 5 min. Then, 150 µL of 10% aluminum chloride (AlCl_3_) was added, and the mixture was vortexed and left to rest for 6 min. Finally, 2000 µL of 4% sodium hydroxide (NaOH) and 1700 µL of distilled water were added, and the mixture was homogenized in a vortex and left to rest at room temperature and protected from light for 15 min. Finally, the absorbance was read at 510 nm using a GENESYS 10 UV-VIS spectrophotometer (Thermo Scientific, Waltham, MA, USA). The calibration curve was generated using quercetin as the standard (at concentrations of 40.0, 80.0, 120.0, 160.0, and 200.0 μg/mL). All the assays were performed in triplicate, and the results are expressed as mg of the quercetin equivalents per g of extract (mg QE/g of extract) [46].

### 4.7. Antioxidant Activity of the Atomized Extract of C. diacanthus

#### 4.7.1. The 2,2-diphenyl 1-picrylhydrazyl (DPPH) Free Radical Scavenging Method

First, 2850 µL of the DPPH working solution was added to 150 µL of the sample (1 mg/mL), which set the absorbance to 0.6 ± 0.02 nm. The sample was left to react in the dark for 30 min, and then it was measured at 517 nm using a GENESYS 10 UV-VIS spectrophotometer [47]. Next, a calibration curve was generated with Trolox as the standard (100–800 μmoles), and the results are expressed as μmol of the Trolox equivalent per gram of extract (μmol TE/g of extract). Then, the inhibitory concentration 50 (IC_50_) of the percentage of inhibition of the DPPH radical was determined using concentrations of 0.25, 0.50, 1.0, and 2.0 mg/mL of the atomized extracts.

#### 4.7.2. Testing Using the 2,2-azinobis-(3-ethylbenzothiazoline)-6-sulfonic Acid (ABTS) Radical Cation Sequestration Method

A volume of 150 µL of the sample (1 mg/mL) was measured and mixed with 2850 µL of the ABTS working solution (ST). The absorbance was set to 0.7 ± 0.02 nm, and the mixture was left to react for 7 min in the dark. Finally, the absorbance was measured at 734 nm using a GENESYS 10 UV-VIS spectrophotometer. The results are expressed in µmol Trolox equivalent/g of extract (µmol TE/g of extract) [48]. In addition, a calibration curve was generated using Trolox as the standard (50–400 μmol), and the results are expressed as μmoles of the Trolox equivalent per gram of extract (μmol TE/g of extract). The inhibitory concentration 50 (IC_50_) of the percentage of inhibition of the ABTS radical was calculated using concentrations of 0.25, 0.50, 1.0, and 2.0 mg/mL of the atomized extracts.

#### 4.7.3. The Ferric-Reducing Antioxidant Power (FRAP)

A volume of 150 µL of the sample (1 mg/mL) was measured, and 2850 µL of the FRAP working solution was added to it. Then, the mixture was left to react for 30 min. Finally, the absorbance was read at 593 nm using a GENESYS 10 UV-VIS spectrophotometer. The results obtained are expressed in µmol of the Trolox equivalent/g of extract (µmol TE/g of extract). Also, a calibration curve was calculated using Trolox as the standard (50–800 μmol) [49]. The inhibitory concentration 50 (IC_50_) was calculated using concentrations of 0.25, 0.50, 1.0, and 2.0 mg/mL of the atomized extracts.

### 4.8. Determination of the Effect of the Atomized Extracts of the Leaves and Stems of Cnidoscolus diacanthus (Pax. & Hoffm.) Macbr. on Hormones in Rats

#### 4.8.1. Animal Conditioning

For this part of the study, 64 Holtzman rats were utilized; these were adult males and females in good health with an average weight between 180 and 220 g. The animals were acquired from the National Institute of Health in Lima, Peru. Subsequently, they were conditioned in breeding cages under a 12 h light–dark cycle at room temperature, and they received food and water ad libitum. This study was approved by the Institutional Ethics Committee, Id. 006-VRI-UNSCH.

#### 4.8.2. Preparation of the Extracts

The atomized leaf and stem extracts were prepared individually at concentrations of 0.25%, 0.50%, and 1.0%. Doses of 25, 50, and 100 mg/kg were orally administered for 18 days [50,51].

#### 4.8.3. Experimental Design

After their acclimatization, the rats were weighed, coded, and allocated according to sex (females and males) into the following groups, with six rats per group:

− Group I: Control group (male rats);
− Group II: Control group (female rats);
− Group III: Atomized leaf extract at a dose of 25 mg/Kg (male rats);
− Group IV: Atomized leaf extract at a dose of 25 mg/Kg (female rats);
− Group V: Atomized leaf extract at a dose of 50 mg/Kg (male rats);
− Group VI: Atomized leaf extract at a dose of 50 mg/Kg (female rats);
− Group VII: Atomized leaf extract at a dose of 100 mg/Kg (male rats);
− Group VIII: Atomized leaf extract at a dose of 100 mg/Kg (female rats);
− Group IX: Atomized stem extract at a dose of 25 mg/Kg (male rats);
− Group X: Atomized stem extract at a dose of 25 mg/Kg (female rats);
− Group XI: Atomized stem extract at a dose of 50 mg/Kg (male rats);
− Group XII: Atomized stem extract at a dose of 50 mg/Kg (female rats);
− Group XIII: Atomized stem extract at a dose of 100 mg/Kg (male rats);
− Group XIV: Atomized stem extract at a dose of 100 mg/Kg (female rats).

#### 4.8.4. Hormonal Evaluation in the Experimental Animals

For quantification of the rats’ sex hormones (FSH, LH, testosterone, estradiol, and progesterone), blood samples were collected by cardiac puncture and processed using a fluorescence-based lateral flow immunoassay in the i-CHROMA III (Boditech Med Inc., Chuncheon-si, Gangwon-do, Republic of Korea).

##### Testosterone Evaluation

To evaluate the rats’ testosterone levels, 30 μL of the displacing reagent was transferred into a sample mixing tube, and 75 μL of serum was added with a micropipette to the tube containing the reagent. The mixture was shaken 10 times and then used immediately. The mixture was then incubated at room temperature for 3 min. Next, 75 μL of the mixture was dispensed into a tube containing the detector buffer, with the sample then mixed completely by shaking it 10 times. Then, 75 μL of the sample was taken from this mixture, dispensed into the well of the cassette, and incubated at room temperature for 12 min before being inserted into the equipment holder. To begin the reading, the cartridge was placed into the i-CHROMA III analyzer. The multi-test option was used, with the reading time set to 3 min intervals for each cassette. The results are given in ng/mL.

##### Progesterone Evaluation

First, 150 µL of detector diluent was transferred into a detector tube containing granules with a micropipette. When the granules had completely dissolved in the detector tube (the detection buffer), 30 µL of serum was placed into the detector tube and mixed about 10 times (the mixture was used immediately within 30 s after shaking it 10 times). Subsequently, 75 µL of the sample mixture was placed in the sample cassette, and it was incubated at room temperature for 15 min. To begin the reading, the cartridge was placed in the i-CHROMA III analyzer. The multi-test option was used, with the reading time set to 3 min intervals for each cassette. The results are expressed in ng/mL.

##### Estradiol Evaluation

Initially, a volume of 25 µL of serum was transferred into the microplate, followed by the addition of 50 µL of the estradiol biotin reagent. The contents were then vigorously mixed for a duration of 20–30 s to ensure thorough homogenization. Then, 50 µL of the estradiol enzyme reagent was added to each well, and the microplate was gently agitated for 20 to 30 s to ensure thorough mixing. Finally, it was incubated for 90 min at room temperature. The contents of the microplate were then decanted and removed. Subsequently, 350 µL of washing buffer was introduced, followed by the addition of 100 µL of substrate solution into the well. The mixture was incubated at room temperature for a duration of 20 min. Each well received 50 µL of the prepared solution, which was then stirred for a duration of 15 to 20 s. The absorbance measurement was taken at a wavelength of 450 nm, with a reference wavelength range of 620–630 nm. The results are reported in ng/mL.

##### FSH Evaluation

A quantity of 75 µL of serum was added to the detection buffer with a micropipette, and the sample was mixed well with the buffer. Then, 75 µL of the mixture was taken and applied to the sample well of the cartridge or cassette and allowed to incubate at room temperature. Then, the cartridge was placed in the i-CHROMA III analyzer. The multi-test option was used, with the reading time set at 1 min intervals for each cassette. The results are expressed in mIU/mL.

##### LH Evaluation

First, 150 μL of serum was transferred into a tube containing the detector buffer with a micropipette, and this was shaken 10 times. Then, 75 µL of the sample mixture was loaded into the sample well of the test cartridge, which was incubated at room temperature for 15 min before being inserted into the holder. To begin reading, the cartridge was placed in the i-CHROMA III analyzer. The multi-test option was used, with the reading time set at 1 min intervals for each cassette. The results are expressed in mIU/mL.

### 4.9. Statistical Analysis

All of the assays for the total phenolic, flavonoid, and antioxidant compounds were performed in triplicate. The results are expressed as the mean ± standard deviation. Differences between the means (using Student’s *t*-test) of the following variables were checked: phenolic compounds, flavonoids, the ability to scavenge the DPPH and ABTS free radicals, and ferric-reducing antioxidant power (FRAP). Differences were considered significant when the *p* value was < 0.05. The hormonal variations in the animals were presented in tables and figures, and statistical evaluation of these results was carried out in a database using Graph Pad Prism program v4.0. Analysis of variance (ANOVA) and Tukey’s test were applied to detect possible differences between the treatments, considering a significance level of 5% (α = 0.05).

## 5. Conclusions

In conclusion, the atomized stem extract was more effective as an antioxidant than the leaf extract in the three tests: DPPH, ABTS, and FRAP. Furthermore, the stems had higher phenol and flavonoid contents than leaves, and these values were associated with more potent antioxidant activities. Regarding the effect on sexual hormones, using 50 mg/kg of the atomized extracts of the leaves and stems of *C. diacanthus* had the best effect in increasing the LH, FSH, E_2_, and progesterone levels in the female rats. For the male rats, the extracts had the opposite effect, an effect potentially relevant to infertility in male rats. However, additional studies on the extracts’ safety and biochemical mechanisms and evaluations of other biochemical sexual markers are needed to extrapolate our findings to human beings. Additionally, the established ethnopharmacological use of this plant in women can be related to these findings because the increase in the hormones studied was more effective in female rats than in male rats.

## Figures and Tables

**Table 1:** Annotated compounds from atomized extract of *C. diacanthus* leaves according to LC–MS analysis.

Assigned Identification	Molecular Formula (*m*/*z*) [M-H]^−^	Retention Time (min)	Product Ions (MS^2^)
Quinic acid [13]	C_7_H_12_O_6_ 191.08	2.04	173 (–H_2_O), 127 (–HCO_2_H), 85 (100%)
Chlorogenic acid (3-O-caffeoylquinic acid) [13,14]	C_16_H_18_O_9_ 352.91	14.62	191 (100%), 179, 173
Neochlorogenic acid (5-O-caffeoylquinic acid) [14]	C_16_H_18_O_9_ 352.81	21.93	191, 179, 173 (100%), 135, 107
Quercetin 3-rhamnoglucoside (rutin) [15]	C_27_H_30_O_16_ 609.01	30.58	463 (–dHex), 301 (100%) (–dHex–Hex = quercetin), 271
Kaempferol 3-O-glucoside-7-O-rhamnoside (nicotiflorin) [15]	C_27_H_30_O_15_ 593.05	32.86	447 (–dHex), 327, 285 (100%) (–dHex–Hex = kaempferol)
Quercetin [16]	C_15_H_10_O_7_ 301.03	39.49	283, 257, 165 (100%), 135, 91
5,6,2′-trimethoxyflavone (N.R.)	C_18_H_16_O_5_ 311.17	53.55	197, 183 (100%), 170, 119

N.R.: non-referenced.

**Table 2:** Annotated compounds from atomized extract of *C. diacanthus* stems according to LC–MS analysis.

Assigned Identification	Molecular Formula (*m*/*z*) [M-H]^−^	Retention Time (min)	Product Ions (MS^2^)
Catechin [17]	C_15_H_14_O_6_ 289.13	19.74	271, 245 (100%), 205, 179
Genistein (N.R.)	C_15_H_10_O_5_ 269.12	48.56	251, 241 (100%), 213, 201
5,6,2-Trimethoxyflavone (N.R.)	C_18_H_16_O_5_ 311.17	53.58	197, 183 (100%), 170, 119

N.R.: non-referenced.

**Table 3:** Contents of total phenols and total flavonoids in the atomized extracts of leaves and stems from *C. diacanthus*.

Atomized Extract	Total Phenols (mg GAE/g Extract)	Total Flavonoids (mg QE/g Extract)	Correlation Flavonoids/Phenols
Leaves	212.38 ± 3.19 *	121.49 ± 2.69 *	0.57
Stems	464.38 ± 4.40 *	369.17 ± 3.16 *	0.79

* The results are the average ± SD of three determinations.

**Table 4:** Trolox-equivalent antioxidant capacity (TEAC) from DPPH, ABTS, and FRAP assays of the atomized extracts of the leaves and stems of *C. diacanthus*.

Antioxidant Assay		Atomized Extract *		Antioxidant Standard	*p* Value (Student’s *t*-Test)
		Leaves	Stems	Trolox	
DPPH	µmol TE/mg extract	462.39 ± 2.0	597.20 ± 2.32	-	*p* < 0.0001
	IC_50_ (µg/mL)	922.04	841.95	3.24 ± 0.01	
ABTS	µmol TE/mg extract	202.32 ± 5.76	452.67 ± 5.20	-	*p* < 0.0001
	IC_50_ (µg/mL)	1396.09	678.70	2.36 ± 0.03	
FRAP	µmol TE/mg extract	198.13 ± 1.44	535.91 ± 1.56	-	*p* < 0.0001

* The results are the average ± SD of three determinations.

## Data Availability

All the relevant data supporting the findings of this study are contained within this article.

## Supplementary Materials

The following supporting information can be downloaded at https://www.mdpi.com/article/10.3390/molecules29194554/s1.
