# Supplementary material for: The Antioxidant Activity of Atomized Extracts of the Leaves and Stems of Cnidoscolus diacanthus (Pax & K. Hoffm.) J.F. Macbr. from Peru and Their Effect on Sex Hormone Levels in Rats
Source: Molecules. 2024 Sep 25;29(19):4554. doi: 10.3390/molecules29194554 (PMC11478110; doi:10.3390/molecules29194554)
Supplement: Supplementary file 1 [file molecules-29-04554-s001.zip › molecules-3180774-supplementary.pdf]

Supplementary Material

The Antioxidant Activity of Atomized Extracts of the Leaves and Stems of *Cnidoscolus diacanthus* (Pax & K. Hoffm.) J.F. Macbr. from Peru and Their Effect on Sex Hormone Levels in Rats

Johnny Aldo Tinco-Jayo [1], León Fernando Pérez-Chauca [2], Nancy Victoria Castilla-Torres [1], Edwin Carlos Enciso-Roca [1], Diana Taboada-Huaman [3], Litman Nuñez-Soto [3], Luis Uriel Moscoso-García [4], Jorge Luis Arroyo-Acevedo [5], Enrique Javier Aguilar-Felices [1] and Oscar Herrera-Calderon [6,*]

[1] Department of Human Medicine, Faculty of Health Sciences, Universidad Nacional de San Cristobal de Huamanga, Portal Independencia 57, Ayacucho 05003, Peru; johnny.tinco@unsch.edu.pe (J.A.T.-J.); nancy.castilla@unsch.edu.pe (N.V.C.-T.); edwin.enciso@unsch.edu.pe (E.C.E.-R.); enri-que.aguilar@unsch.edu.pe (E.J.A.-F.)
[2] Academic Department of Chemical Engineering, Universidad Nacional de San Cristóbal de Huamanga, Portal Independencia 57, Ayacucho 05003, Peru; leon.perez@unsch.edu.pe
[3] Professional School of Pharmacy and Biochemistry, Faculty of Health Sciences, Universidad Nacional de San Cristobal de Huamanga, Portal Independencia 57, Ayacucho 05003, Peru; di-ana.taboada.20@unsch.edu.pe (D.T.-H.); litman.nunez.20@unsch.edu.pe (L.N.-S.)
[4] Academic Department of Biological Sciences, Faculty of Biological Sciences, Universidad Nacional de San Cristobal de Huamanga, Portal Independencia 57, Ayacucho 05003, Peru; luis.moscoso@unsch.edu.pe
[5] Department of Dynamic Sciences, Faculty of Medicine, Universidad Nacional Mayor de San Marcos, Av. Miguel Grau 755, Lima 15001, Peru; jarroyoa@unmsm.edu.pe
[6] Department of Pharmacology, Bromatology and Toxicology, Faculty of Pharmacy and Biochemistry, Universidad Nacional Mayor de San Marcos, Jr. Puno 1002, Lima 15001, Peru

* Correspondence: oherreraca@unmsm.edu.pe; Tel. +51-956550510

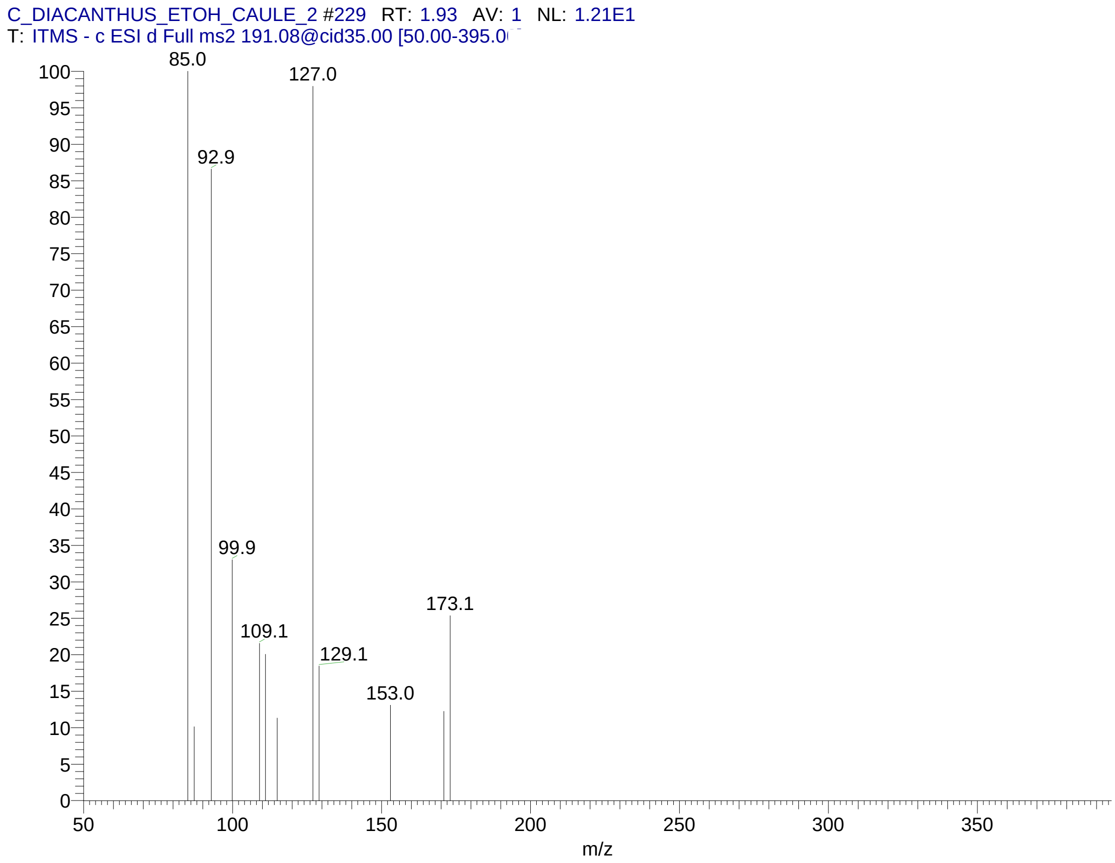

Figure S1. Product ion spectra of quinic acid.

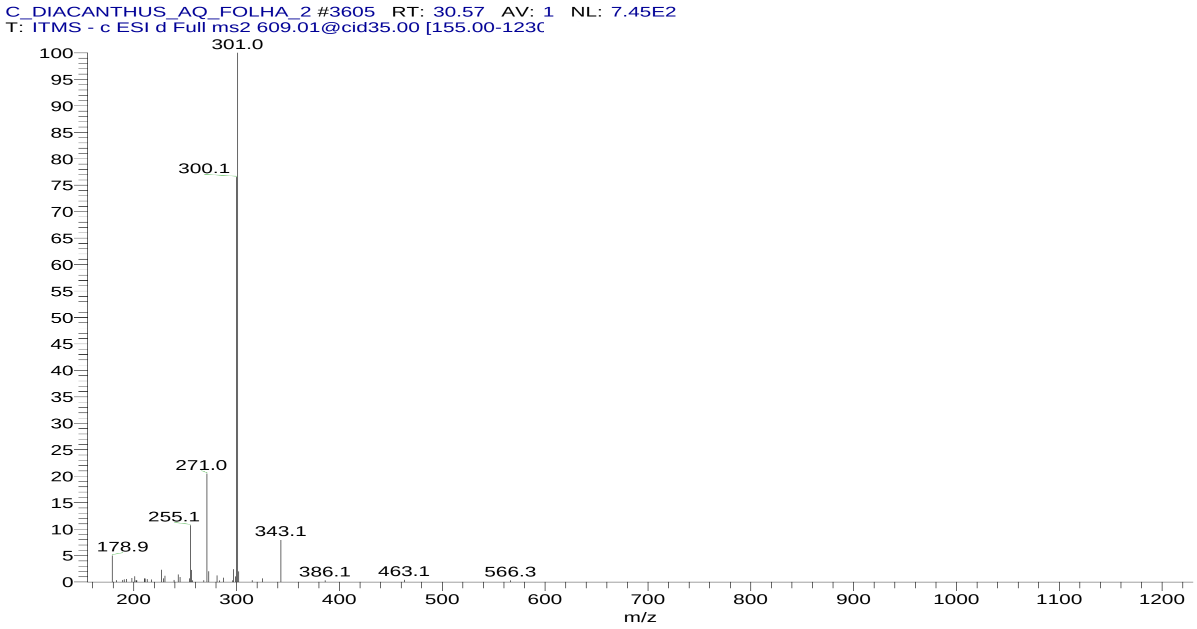

Figure S2. Product ion spectra of rutin.

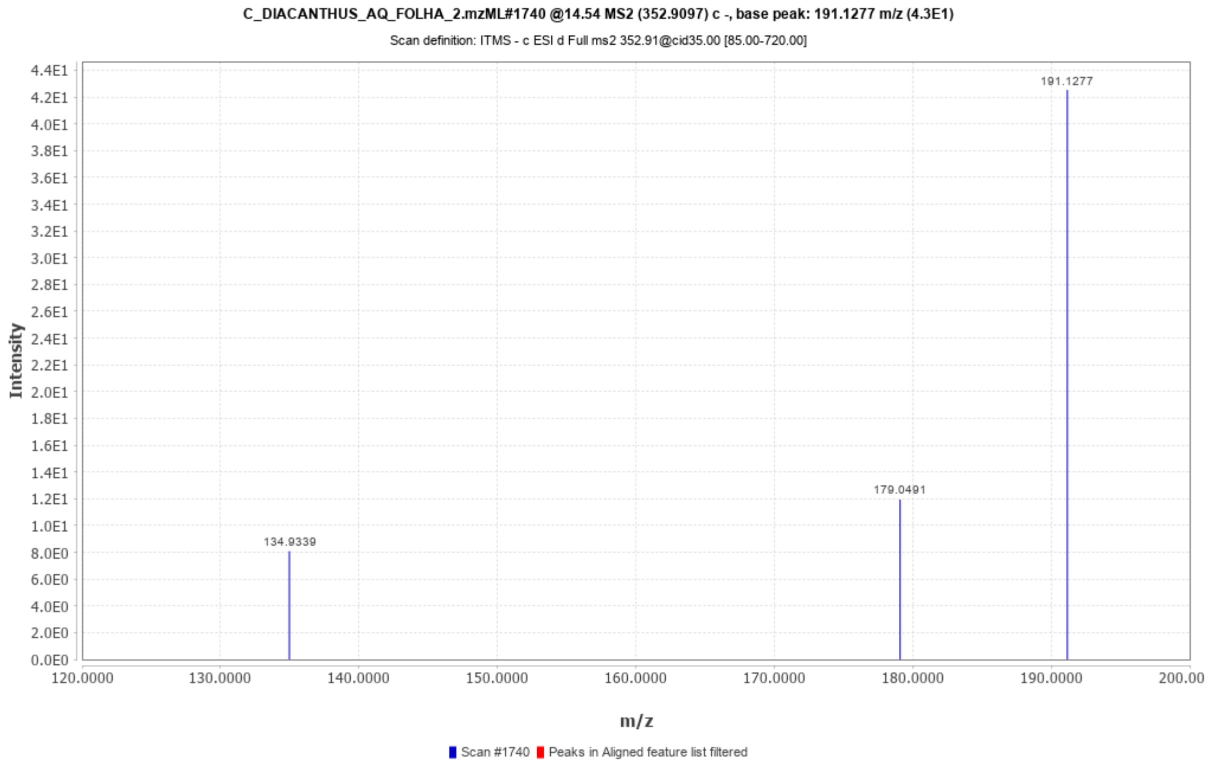

Figure S3. Product ion spectra of chlorogenic acid.

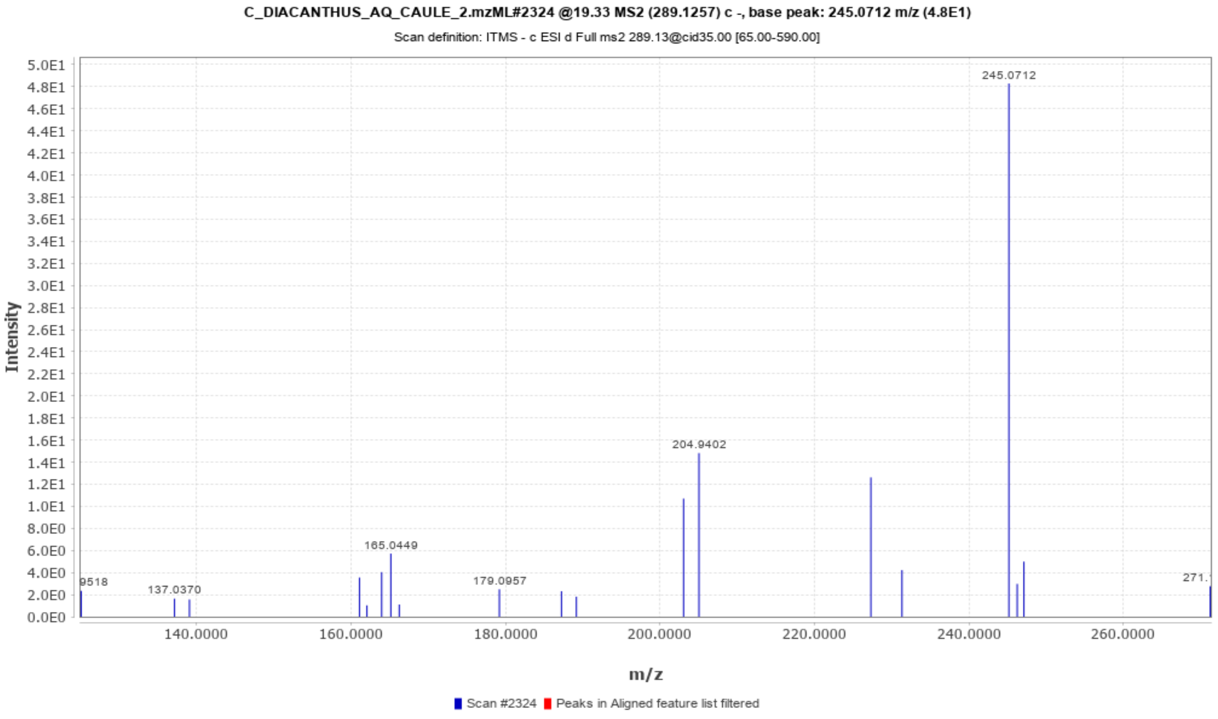

Figure S4. Product ion spectra of catechin.

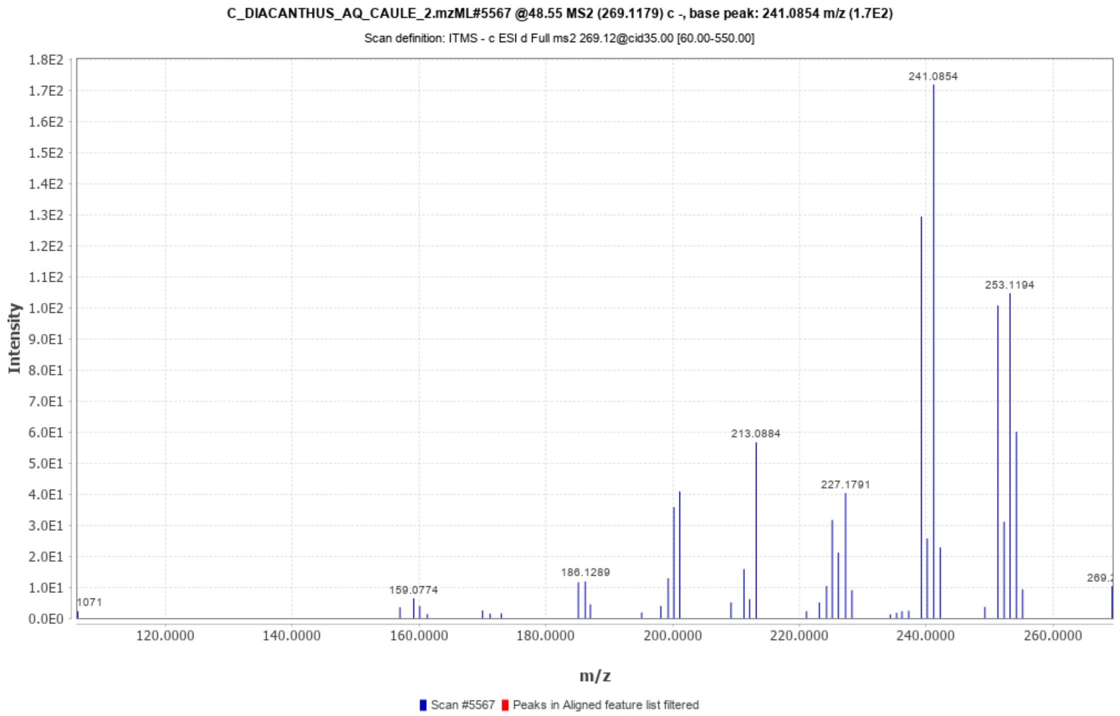

Figure S5. Product ion spectra of genistein.

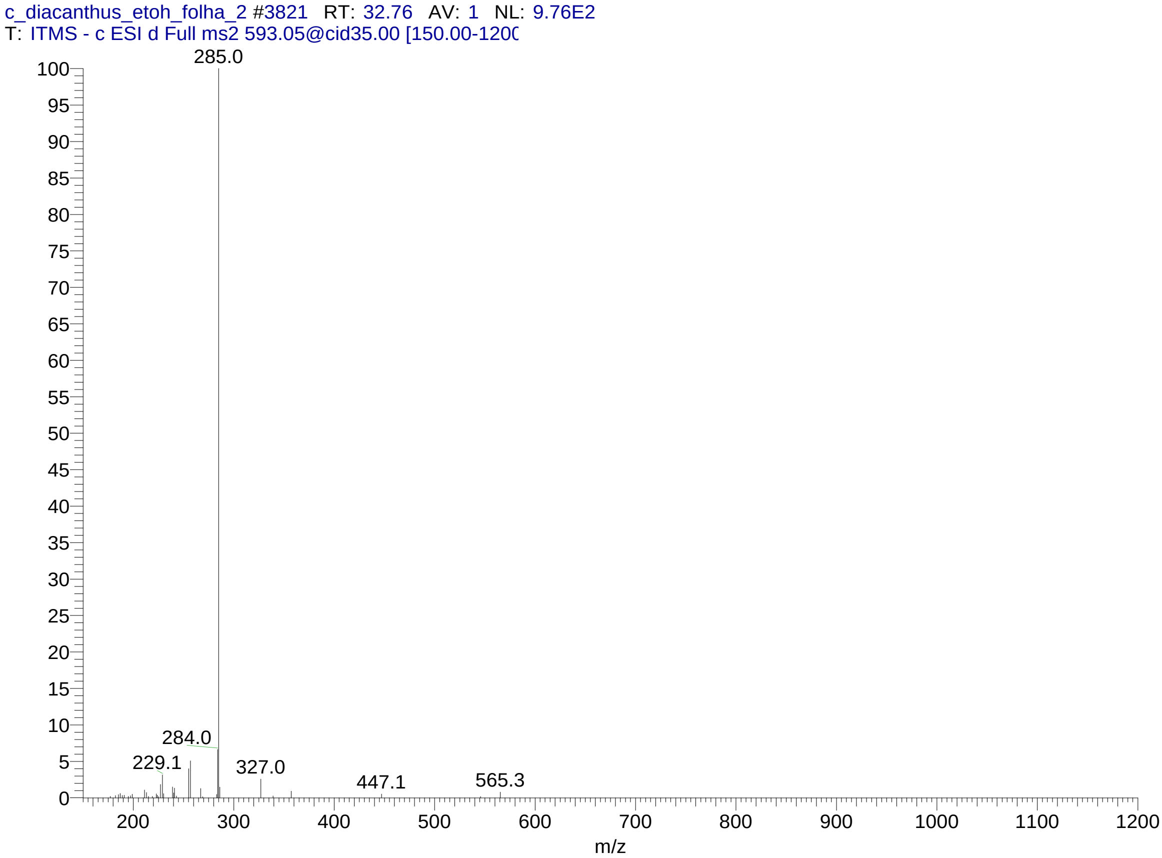

Figure S6. Product ion spectra of Kaempferol-3-O-glucoside-7-O-rhamnoside.

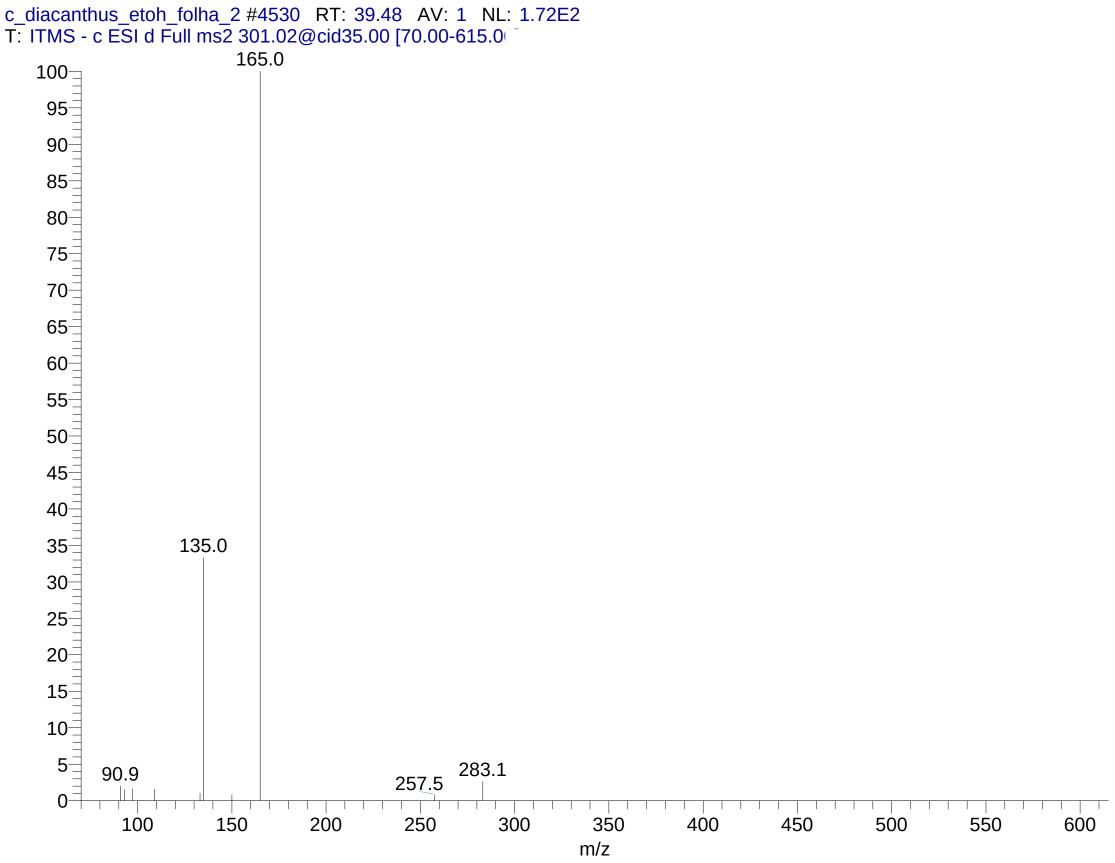

Figure S7. Product ion spectra of quercetin.

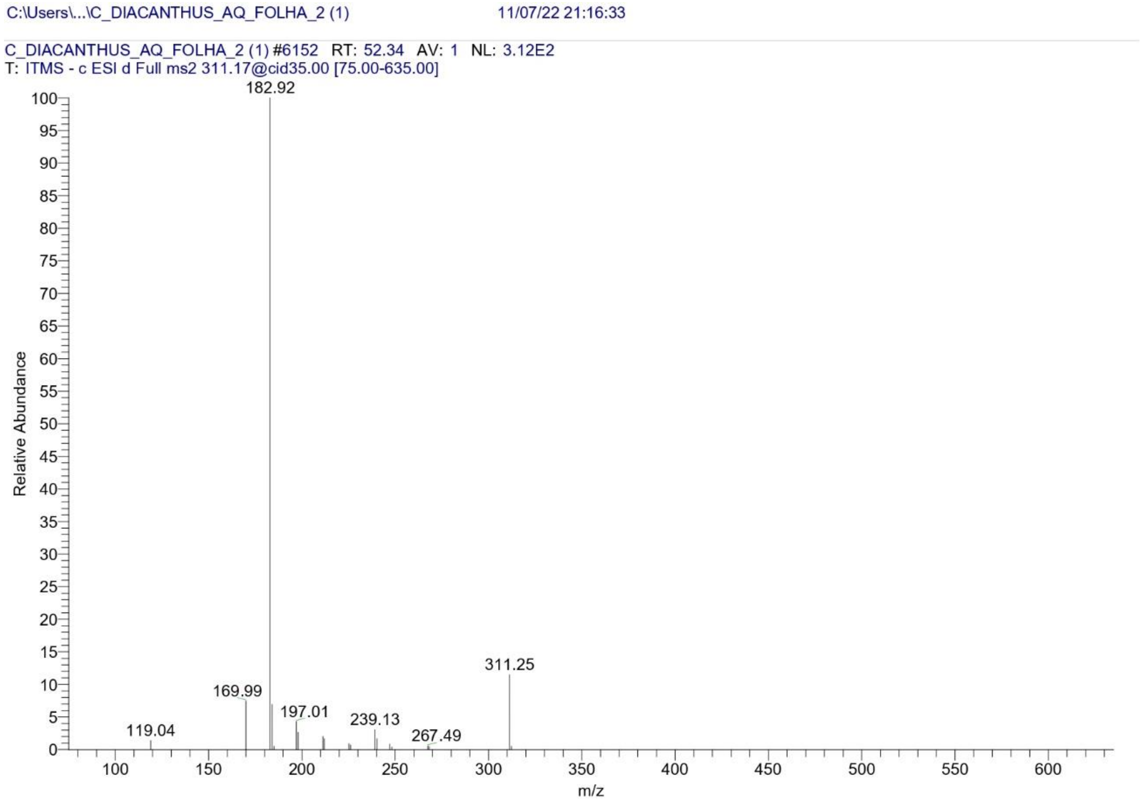

Figure S8. Product ion spectra of 5,6,2'-trimethoxyflavone.
